# Decentralized System Identification Using Stochastic Subspace Identification for Wireless Sensor Networks

**DOI:** 10.3390/s150408131

**Published:** 2015-04-08

**Authors:** Soojin Cho, Jong-Woong Park, Sung-Han Sim

**Affiliations:** 1 School of Urban and Environmental Engineering, Ulsan National Institute of Science and Technology (UNIST), Ulsan 689-798, Korea; E-Mail: soojin@unist.ac.kr; 2 Department of Civil and Environmental Engineering, University of Illinois at Urbana-Champaign, Urbana, IL 61801, USA; E-Mail: smart.jwp@gmail.com

**Keywords:** wireless sensor, structural health monitoring, decentralized processing, system identification, stochastic subspace identification

## Abstract

Wireless sensor networks (WSNs) facilitate a new paradigm to structural identification and monitoring for civil infrastructure. Conventional structural monitoring systems based on wired sensors and centralized data acquisition systems are costly for installation as well as maintenance. WSNs have emerged as a technology that can overcome such difficulties, making deployment of a dense array of sensors on large civil structures both feasible and economical. However, as opposed to wired sensor networks in which centralized data acquisition and processing is common practice, WSNs require decentralized computing algorithms to reduce data transmission due to the limitation associated with wireless communication. In this paper, the stochastic subspace identification (SSI) technique is selected for system identification, and SSI-based decentralized system identification (SDSI) is proposed to be implemented in a WSN composed of Imote2 wireless sensors that measure acceleration. The SDSI is tightly scheduled in the hierarchical WSN, and its performance is experimentally verified in a laboratory test using a 5-story shear building model.

## 1. Introduction

As deteriorating civil infrastructure has received considerable public attention, structural health monitoring (SHM) has become widely employed to maintain the structures sound construction and prevent catastrophic collapses. Data acquisition in traditional SHM systems is based on wired sensors connected to a centralized data collection repository. All sensor data is aggregated at this central repository, where all data processing takes place to extract structural features and information. The centralized data acquisition and processing approach in the wired sensor network is the common practice in traditional SHM systems; however, high cost and installation difficulties [1] have prevented the SHM from wider adoption in large-scale civil structures. For instance, to install a wired connection-based SHM system on the Golden Gate Bridge, miles of cables would be required to connect the central base station to a dense array of sensor nodes distributed along the deck, towers, and cables; installation would be both costly and time-consuming, and maintenance of the system would be challenging.

Wireless sensor networks (WSNs) provide a promising alternative to the traditional SHM approach. Wireless sensors commonly refer to sensors that are small, inexpensive, capable of wireless communication, and have on-board processing capabilities [2]. In the last decades, many academic and commercial wireless sensors have been developed. Significant efforts have been devoted to various issues in WSNs, including data acquisition, processing, and damage detection. The majority of wireless sensor research has focused on emulation of traditional wired sensor networks employing centralized data acquisition and processing [3,4,5]. Such approaches have proven to be intractable because transferring all sensor data quickly saturates the limited bandwidth in wireless communication and thus causes severe network congestion. Indeed, decentralized data processing schemes are considered to be essential to ensure the scalability of WSNs required to enable a dense array of sensors deployed on full-scale civil structures.

One of the main decentralized data processing approaches is the coordinated processing on hierarchical networks [6]. Nagayama and Spencer [7] implemented the coordinated processing using NExT/ERA (Natural Excitation Technique/Eigensystem Realization Algorithm) [8,9] on their WSN for damage detection. Furthermore, frequency-domain decomposition (FDD) [10] and random decrement technique (RDT) [11] have also been used for system identification purposes in WSNs. Sadhu *et al.* [12] used the blind source separation technique for decentralized modal identification of a pony truss pedestrian bridge using a WSN. Wu *et al.* [13] realized a decentralized WSN using a Hilbert-Huang transform for structural health monitoring of an in-lab cable-stayed bridge model. More decentralized approaches have been intensively studied, being proven to be efficient from both computation and wireless communication perspectives [14,15,16].

Stochastic subspace identification (SSI) technique has the potential to enhance the ability for decentralized system identification. In particular, SSI with the canonical variate algorithm (CVA) weighting (SSI/CVA) normalizes the natural modes in terms of energy; thus, less excited modes can be better identified [17]. In the comparative study of various system identification methods, Yi and Yun [18] have shown the SSI/CVA estimates dynamic properties more accurately than peak picking, FDD, and NExT/ERA with a single reference.

This study presents a decentralized system identification using the SSI/CVA. The approach is implemented on the Imote2-based WSN and experimentally verified on a 5-story shear building model. In addition, the proposed SSI-based decentralized system identification is compared to the NExT/ERA-based one in terms of the accuracy and data transmission. Note that, hereinafter, the SSI/CVA is referred to as SSI for simplicity.

## 2. Decentralized System Identification in WSN

Decentralized in-network data processing has been introduced to resolve the problems in the traditional centralized data acquisition and processing in WSNs such as data inundation and network congestion. In decentralized data processing, each sensor in the network participates in processing measured data to extract meaningful information such as dynamic properties (*i.e.*, natural frequencies, damping factors, and mode shapes) and damage identification results. As the extracted information is much smaller than the raw sensor data in size, collecting this information only can reduce wireless data transmission.

### 2.1. Decentralized Data Processing Schemes

**Figure 1:**
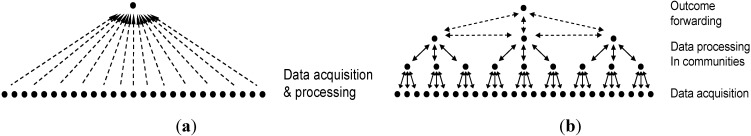
Data acquisition and processing schemes [7]. (**a**) Decentralized independent processing; (**b**) Decentralized coordinated processing.

One of the decentralized approaches uses independent processing [19,20,21,22,23,24,25,26] as shown in Figure 1. Each of the sensor node processes measured data independently without communicating with other sensor nodes. The processed data, typically fast Fourier transform (FFT) or signature analysis, is then sent to the base station. The total amount of data transferred over the radio in the network is quite small. Although sensor networks based on this processing scheme are scalable, important spatial information (e.g., mode shape) cannot be extracted.

The decentralized approach proposed by Gao *et al.* [6] employs a coordinated computing strategy, often called distributed computing strategy (DCS), as shown in Figure 1b, which has the ability to capture local spatial information. The sensor network in this scheme is divided into hierarchical communities, in which sensor nodes within each community communicate with each other in processing data; communication between communities is conducted through each community’s cluster-head. Nagayama and Spencer [7] implemented the DCS logic in a WSN employing Imote2 wireless sensors. NExT/ERA was implemented in the network for the community-wide data processing to identify modal properties of the local community.

### 2.2. Stochastic Subspace Identification

This study explores the use of SSI in the community-wide data processing in WSNs. For completeness, SSI is briefly reviewed. Consider a discrete time stochastic state space model:

(1) $$\begin{array}{c} x_{k+1}=Ax_{k}+w_{k}\\ y_{k}=Cx_{k}+v_{k} \end{array}$$

where $w_{k}$ and $v_{k}$ are uncorrelated zero mean white noise vectors, $x_{k}$ is the n×1 state vector, and $y_{k}$ is the m×1 output vector . The correlation function $R_{k}$ of the output sequence can be expressed as:

(2) $$R_{k}=E\left[y_{k+l}y_{l}^{T}\right]=CA^{k-1}G$$

where $G=E[x_{k+1}y_{k}^{\text{T}}]$. The block Hankel matrix $H_{p,q}$ with p block rows and q block columns, composed of the correlation function $R_{k}$, can be decomposed as:

(3) $$H_{p,q}=\left[\begin{array}{ccc} R_{1} & \cdots & R_{q}\\ \vdots & \ddots & \vdots \\ R_{p} & \cdots & R_{p+q-1} \end{array}\right]=O_{p}C_{q}$$

where $O_{p}=\left[\begin{array}{c} C\\ CA\\ \vdots \\ CA^{p-1} \end{array}\right]$ and $C_{q}=\left[\begin{array}{cccc} G & AG & \cdots & A^{q-1}G \end{array}\right]$. $O_{p}$ and $C_{q}$ are the observability and extended controllability matrices, respectively. The weighted block Hankel matrix calculated by pre- and post-multiplying weighting matrices $W_{1}$ and $W_{2}$ becomes:

(4) $$W_{1}H_{p,q}W_{2}=W_{1}O_{p}C_{q}W_{2}=\left[\begin{array}{cc} U_{1} & U_{2} \end{array}\right]\left[\begin{array}{cc} S_{1} & 0\\ 0 & 0 \end{array}\right]\left[\begin{array}{c} V_{1}^{T}\\ V_{2}^{T} \end{array}\right]=U_{1}S_{1}V_{1}^{T}$$

One possible solution to Equation (4) is

(5) $$O_{p}=W_{1}^{-1}U_{1}S_{1}^{1/2}$$

Then, the system matrices A and C can be readily obtained using Equation (3) with the known observability matrix $O_{p}$ in Equation (5). The system matrix A can be calculated by solving the following equation.

(6) $$O_{p-1}^{↑}=O_{p-1}A$$

where $O_{p-1}^{↑}=\left[\begin{array}{c} CA\\ CA^{2}\\ \vdots \\ CA^{p-1} \end{array}\right]$ and $O_{p-1}=\left[\begin{array}{c} C\\ CA\\ \vdots \\ CA^{p-2} \end{array}\right]$.

The system matrix C is simply the first m rows of $O_{p}$. Depending on the weighting matrices, SSI is called balanced realization (BR) if no weighting matrices are employed, or CVA if the weighting matrices $W_{1}$ and $W_{2}$ are defined as:

(7) $$W_{1}={\left(L^{+}\right)}^{-1}\text{ and }W_{2}={\left(L^{-}\right)}^{-1}$$

where $R^{+}=\left[\begin{array}{ccc} R_{0} & \cdots & R_{p-1}^{T}\\ \vdots & \ddots & \vdots \\ R_{p-1} & \cdots & R_{0} \end{array}\right]=L^{+}L^{+T}$ and $R^{-}=\left[\begin{array}{ccc} R_{0} & \cdots & R_{q-1}^{T}\\ \vdots & \ddots & \vdots \\ R_{q-1} & \cdots & R_{0} \end{array}\right]=L^{-}L^{-T}$.

To form the weighting matrices, the correlation function $R_{k}$ is a square matrix with all combinations of measured signals used in calculating $R_{k}$. As opposed to NExT/ERA that requires correlation functions with respect to a single signal, the use of SSI in WSNs causes more data transmission than NExT/ERA-based system identification. More details are discussed in the following sections.

## 3. Decentralized Network

**Figure 2:**
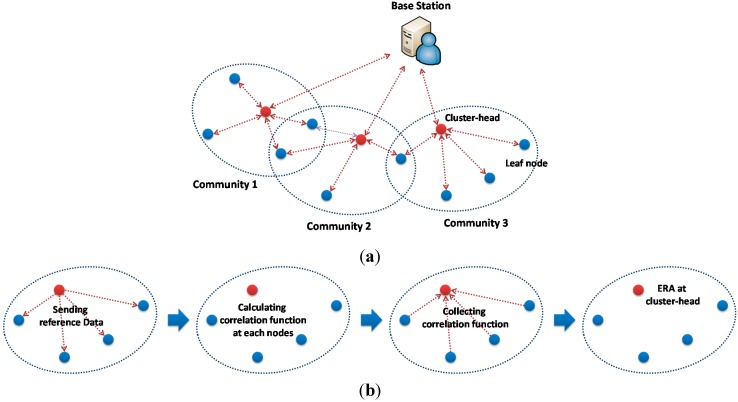

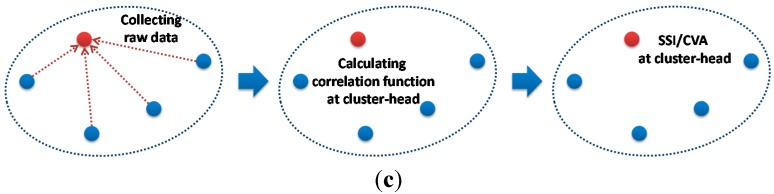
Decentralized coordinated processing. (**a**) Network topology; (**b**) Community-wide data processing: decentralized; (**c**) Community-wide data processing: centralized.

Organizing data transmission and processing appropriately is important to effectively implement SSI for community-wide data processing. Nagayama and Spencer [7] proposed a NExT-based data processing approach (see Figure 2a,b). In this scheme, the whole sensor network is divided into local sensor communities, each of which consists of one cluster-head and multiple leaf nodes. The sensor network initially conducts synchronized sensing and, subsequently, the local sensor communities start processing the data. In each sensor community, the cluster-head sends a time history of sensor data to all leaf nodes in the community as the reference information. Each leaf node and the cluster-head calculate correlation functions between their own measured data and the received/sent reference data. The correlation functions are subsequently collected at the cluster-head. Jang *et al.* [27] used the collected correlation functions in ERA to obtain the modal properties for damage detection. Because the correlation function is generally much shorter than the raw sensor data, the total amount of wireless data transmission can be significantly reduced.

This approach requires the reference signal transmitted from the cluster-head, which is however inefficient for SSI in terms of wireless communication. Because a full correlation function matrix is necessary for SSI as described previously, all sensor nodes should send measured data as reference signals to other sensor nodes as well as correlation functions to the cluster-head after the calculation. Alternatively, this study employs the community-wide centralized data collection and processing approach (see Figure 2c).

The centralized community-wide data processing simply collects all sensor data from leaf nodes and processes the data. The full correlation function matrix is estimated and used as the input of SSI. Although the cluster-head spends much more power on the calculation and, thus, the battery drains quickly in the particular node, this problem can be easily solved by selecting different cluster-heads based on the battery level whenever running the community-wide data processing. While the network-wide centralized data collection is not scalable and is, thus, undesirable for dense sensor networks, the hierarchical network with the community-wide centralized approach is still scalable if the size of each community is kept small and independent related to the size of the total network.

## 4. Implementation

The decentralized data processing scheme employing SSI is implemented on the Imote2-based sensor network. As SSI is computationally demanding, the hardware performance of the sensor platform is crucial. This section describes a brief overview of the Imote2 sensor platform and the implementation of the proposed approach on the sensor network using Imote2.

### 4.1. Imote2 Sensor Platform

**Figure 3:**
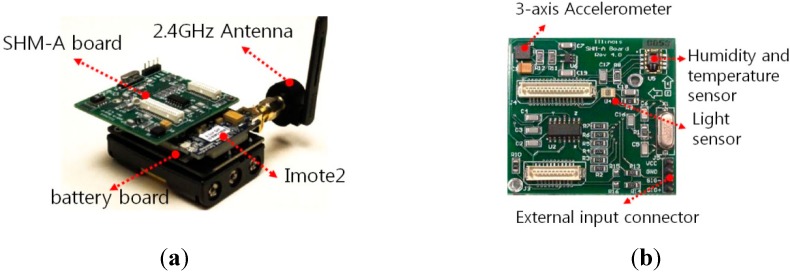
Hardware components of wireless sensor node. (**a**) Imote2; (**b**) ISM400 Board.

Imote2 shown in Figure 3 is a high-performance wireless, computing module. Imote2 has PXA271 XScale^®^ processor (Intel, Santa Clara, CA, USA) running at 13–416 MHz and an MMX DSP coprocessor with memory spaces of 256kB SRAM, 32MB FLASH, and 32MB SDRAM. These powerful processors and large amounts of memory space enable long-term measurement as well as on-board processing of large data. In addition, Imote2 uses 2.4GHz wireless communication with either on-board or external antenna.

As shown in Figure 3, Imote2 can be interfaced with sensor boards that can measure data such as acceleration, strain, temperature, humidity, and light, depending on the attached sensor boards. As acceleration is required in this study, ISM400 sensor boards developed at the University of Illinois at Urbana-Champaign [28] is used. The ISM400 board has a 3-axis accelerometer (LIS344ALH, ST Microelectronic, Geneva, Switzerland) and the embedded QF4A512 (Quickfilter, Dallas, TX, USA) to convert measured analog data to digital signals. QF4A512 has a 4-channel, 16-bit analog to digital converter (ADC) and a programmable signal conditioner with user-selectable sampling rates and programmable digital filters. The ISM400 board also contains temperature, humidity, and light sensors.

### 4.2. Implementation on Imote2

The decentralized system identification using SSI on the Imote2-based sensor network is implemented using the Illinois SHM Project (ISHMP) [29] services a toolsuite. The toolsuite is developed by the University of Illinois at Urbana-Champaign to provide open source middleware services and applications that implement essential components in WSNs such as time synchronization, synchronized sensing, reliable wireless communication, and a wide variety of numerical libraries. These fundamental components can be used as building blocks in developing new WSN applications, dramatically reducing the time and effort necessary for the development. More detailed information regarding the ISHMP Services Toolsuite can be found in [30].

The WSN application, *SSI-based Decentralized System Identification* (SDSI), is developed using the ISHMP Services Toolsuite. SDSI performs SSI to identify system information (*i.e.*, state space representation and modal properties) in each local sensor community, implementing the community-wide centralized data collection and processing. Software components of the toolsuite used in the development include:

1. *Time Synchronization* to synchronize local clocks in each sensor node
2. *Unified Sensing* for measuring acceleration
3. *SensingUnit* that is a high-level service that utilizes Time Synchronization and Unified Sensing services to perform network-wide sensing
4. *ReliableComm* for reliable wireless communication
5. *RemoteCommand* for sensor nodes to interact with each other in a way that command messages are conveyed to receiver nodes that perform designated tasks such as sensing and computing.

Note that the italics denote names of software components in the ISHMP Services Toolsuite. As these basic components are provided by the ISHMP Services Toolsuite, the development is focused on implementing the community-wide data collection, estimation of the full correlation function matrix, and SSI.

**Figure 4:**
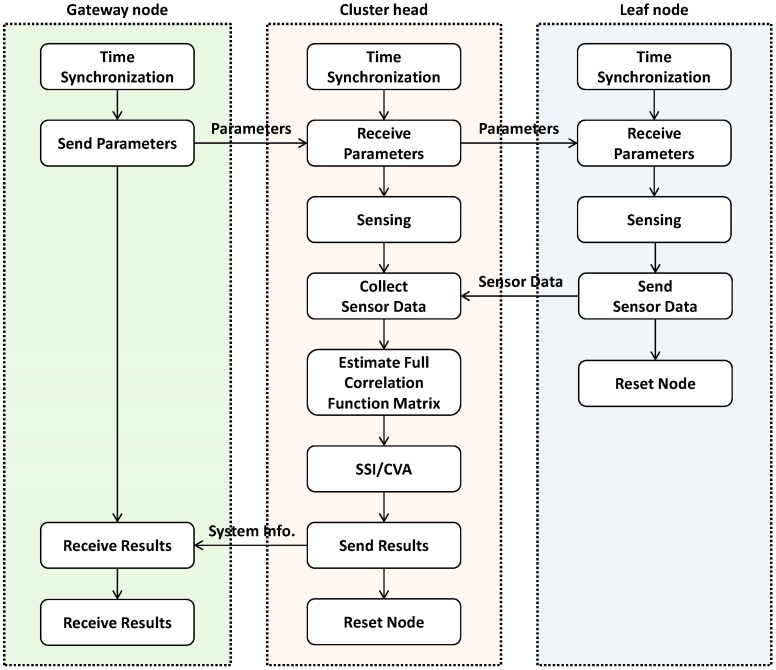
Flowchart of SDSI.

The flowchart in Figure 4 shows how the network is organized for SDSI to produce system identification results from each sensor community. The WSN consists of three types of nodes: (1) a gateway node that is attached to the base station computer and controls the operation of the WSN; (2) cluster-heads that estimate correlation function and system information of their local sensor communities; and (3) leaf nodes that provide sensor data to the cluster-head. As shown in Figure 4, the WSN conducts the network-wide time synchronization and, subsequently, the gateway node disseminates parameters such as the sampling frequency and sensor topology. With received parameters, sensor nodes (*i.e.*, cluster-heads and leaf nodes) measure acceleration data, which are all centrally collected at the cluster-heads in each community. The cluster-heads use the collected data to estimate the full correlation function matrices that are used in SSI. The calculated system information of each community is sent to the gateway node and the base station where the network administrators and developers can access the collected information.

As such, the developed SDSI is able to estimate the system information as the form of the state space representation and modal properties in each sensor community. Due to the decentralized nature of SDSI, the output spatial information is not complete but the collection of partial information from different communities. This partial information can be combined together if overlapping nodes between communities are present [31].

## 5. Experimental Validation

The performance of SDSI is experimentally investigated using the 5-story shear building model. From the random vibration testing, modal properties are estimated using the decentralized in-network processing of SDSI and compared to those obtained based on the centralized data collection and processing. This section describes the experimental setup and system identification results from SDSI.

### 5.1. Experimental Setup

**Figure 5:**
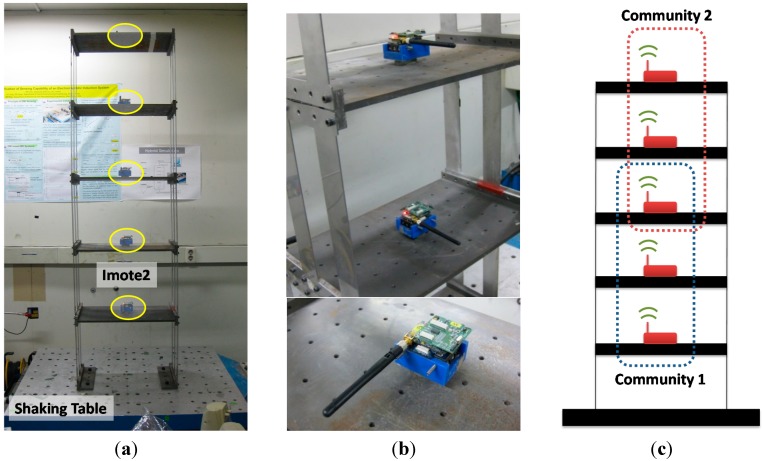
Experimental setup. (**a**) Shear building; (**b**) Installed Imote2; (**c**) Network topology.

The test bed is the shear building model with 6 Imote2 sensor nodes on each floor (see Figure 5). The shear building is flexible in the horizontal direction while relatively rigid in the transverse direction; thus, bending modes in the transverse direction and torsional modes are not expected in the low frequency region. The shaking table in Figure 5a is used to horizontally excite the test bed with a band-limited white noise on the interval 0–15 Hz so that the horizontal bending modes are well excited. The Imote2 sensor nodes shown in Figure 5b measure horizontal accelerations with a sampling rate of 50 Hz associated with a 20 Hz cutoff frequency, which is sufficient to capture the lower five modes. Note that the ISM400 sensor board supports up to 280 Hz for sampling. Each acceleration signal is 10,752 points in length, allowing 20 times of averaging with the number of FFT of 1024 and 50% overlap between spectral windows.

**Figure 6:**
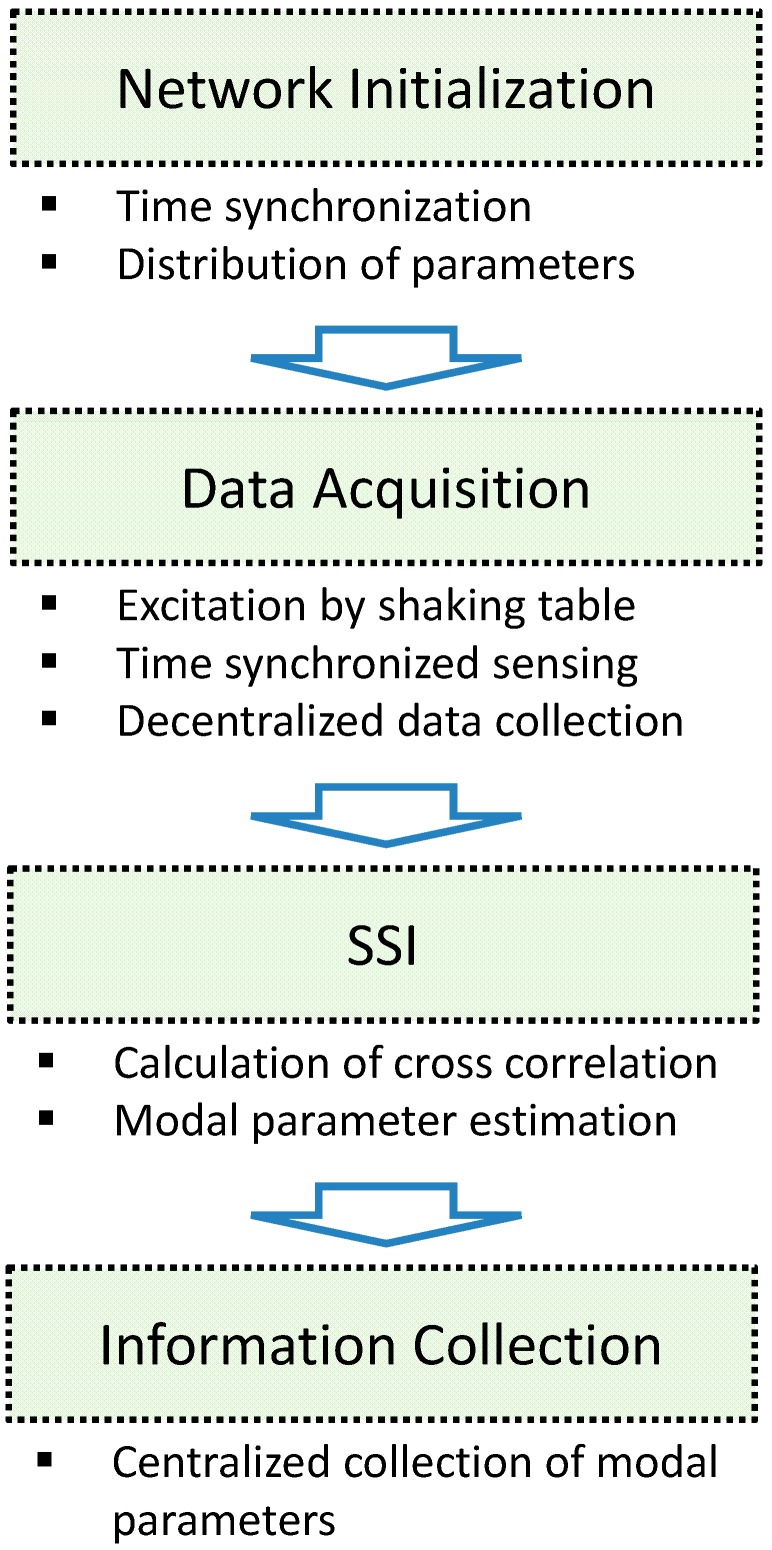
Flowchart of the experiment.

Two local sensor communities are considered as shown in Figure 5c to apply the decentralized in-network data processing using SDSI. Each sensor network has three sensor nodes and shares the node in the 3rd floor. Due to this overlapping node, local mode shapes from communities 1 and 2 can be combined to produce the global mode shapes. Nodes in the 1st and 5th floors are designated to cluster-heads. The experiment is conducted following the procedure shown in Figure 6.

### 5.2. Experimental Results

The SDSI is used to perform decentralized in-network data processing using SSI in the random vibration testing. As designed, the network of Imote2 sensors measures horizontal acceleration, estimates correlation functions, and applies SSI. Local modal properties from two cluster-heads are collected at the base station and assembled to yield global modal properties. The natural frequencies of the whole system can be reasonably determined as the mean values of the natural frequencies from two local communities, as the natural frequency is in general a global property. Local mode shapes can be connected to each other, as two local communities share a sensor node.

**Figure 7:**
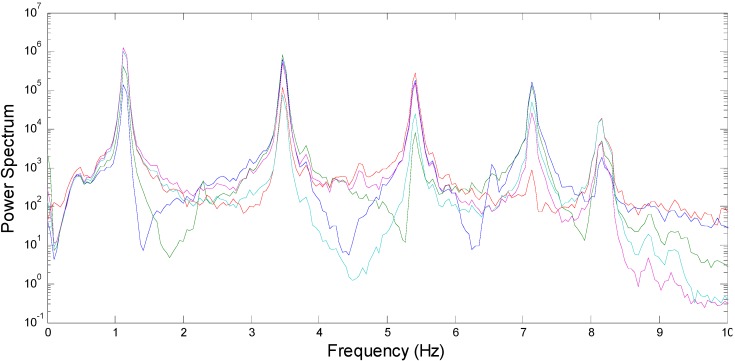
Power spectra of measured acceleration responses.

To verify the results obtained from the decentralized data processing, the centralized approach is employed as the reference information: all measured acceleration time histories are collected at the base station and used to calculate the modal properties using MATLAB. In addition, the power spectra of the collected accelerations shown in Figure 7 commonly have five peaks expected to correspond to five bending modes in the horizontal directions.

To quantitatively evaluate the accuracy with respect to the reference, the difference ratio $E_{f}$ in Equation (8) for natural frequencies and the modal assurance criterion (MAC) [32] in Equation (9) for mode shapes are selected as accuracy measures.

(8) $$E_{f}(\%)=\frac{f_{d}-f_{c}}{f_{c}}\times 100$$

(9) $$\text{MAC}(\phi_{1},\phi_{2})=\frac{\left|\phi_{1}\cdot \phi_{2}\right|}{\left|\phi_{1}\right|\left|\phi_{2}\right|}$$

**Figure 8:**
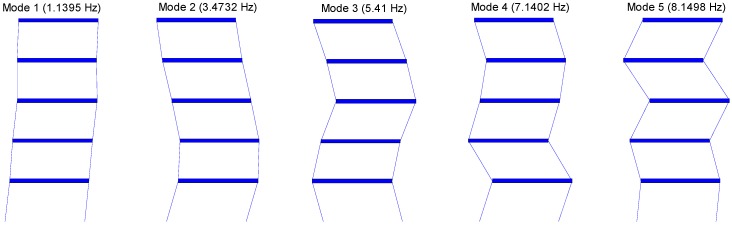
Identified natural frequencies and corresponding mode shapes.

Note that $E_{f}$ and MAC close to 0 and 1, respectively, represent the accurate estimation. Using $E_{f}$ and MAC, the modal properties estimated from the WSN using the in-network data processing are compared to those calculated on the PC in the centralized way as shown in Table 1. $E_{f}$ is less than 0.2% and MAC is close to 1 for all modes; SDSI is able to consistently produce accurate system identification results. Additionally, the combined global mode shapes based on the SDSI results are shown in Figure 8. As such, the decentralized system identification using SSI is successfully implemented on the WSN employing the Imote2 sensor nodes.

**Table 1:** Comparison of modal properties from centralized and decentralized data processing schemes.

Mode Order	Natural Frequency			Mode Shape
	Centralized ($f_{c}$)	Decentralized ($f_{d}$)	$E_{f}(\%)$	MAC
1	1.139	1.140	0.02	1.00
2	3.474	3.473	0.02	1.00
3	5.407	5.410	0.06	0.99
4	7.134	7.140	0.09	0.99
5	8.135	8.150	0.19	0.99

## 6. Conclusions

This study presented the decentralized system identification using SSI for WSNs. The proposed approach is based on the hierarchical network that consists of local sensor communities. Once structural responses are measured by the network-wide synchronized sensing as the first step, each sensor community starts the community-wide data processing: the cluster-head centrally collects sensor data from all leaf nodes in their community and estimates the full correlation functions matrices that are subsequently used as the input to SSI. The calculated local system information from each community is sent to the base station to obtain the global information. This process is implemented on the Imote2-based WSN using the ISHMP Services Toolsuite.

The decentralized system identification with SSI and its implementation on the WSN were experimentally verified using the 5-story shear building model. A total of five Imote2 sensors were placed on each floor and measured acceleration responses due to the base excitation. The network is divided into two local sensor communities, each of which has three Imote2 sensor nodes and shares one node with the other community. The identification results from the WSN and the centralized approach are close to each other with *E_f_* less than 0.2% and MAC of 1 or 0.99, showing the validity of the proposed approach.
